# Genetic Diversity in Endangered Guizhou Snub-Nosed Monkeys (*Rhinopithecus brelichi*): Contrasting Results from Microsatellite and Mitochondrial DNA Data

**DOI:** 10.1371/journal.pone.0073647

**Published:** 2013-08-29

**Authors:** Jakob Kolleck, Mouyu Yang, Dietmar Zinner, Christian Roos

**Affiliations:** 1 Primate Genetics Laboratory, German Primate Center, Leibniz Institute for Primate Research, Göttingen, Germany; 2 Fanjingshan National Nature Reserve, Jiangkou, China; 3 Cognitive Ethology Laboratory, German Primate Center, Leibniz Institute for Primate Research, Göttingen, Germany; 4 Gene Bank of Primates, German Primate Center, Leibniz Institute for Primate Research, Göttingen, Germany; Natural History Museum of Denmark, Denmark

## Abstract

To evaluate the conservation status of a species or population it is necessary to gain insight into its ecological requirements, reproduction, genetic population structure, and overall genetic diversity. In our study we examined the genetic diversity of Rhinopithecus brelichi by analyzing microsatellite data and compared them with already existing data derived from mitochondrial DNA, which revealed that *R. brelichi* exhibits the lowest mitochondrial diversity of all so far studied *Rhinopithecus* species. In contrast, the genetic diversity of nuclear DNA is high and comparable to other *Rhinopithecus* species, i.e. the examined microsatellite loci are similarly highly polymorphic as in other species of the genus. An explanation for these differences in mitochondrial and nuclear genetic diversity could be a male biased dispersal. Females most likely stay within their natal band and males migrate between bands, thus mitochondrial DNA will not be exchanged between bands but nuclear DNA via males. A Bayesian Skyline Plot based on mitochondrial DNA sequences shows a strong decrease of the female effective population size (N_ef_) starting about 3,500 to 4,000 years ago, which concurs with the increasing human population in the area and respective expansion of agriculture. Given that we found no indication for a loss of nuclear DNA diversity in R. brelichi it seems that this factor does not represent the most prominent conservation threat for the long-term survival of the species. Conservation efforts should therefore focus more on immediate threats such as development of tourism and habitat destruction.

## Introduction

Many animal species are threatened by extinction and for a number of species long-term survival is questionable without a proper conservation and population management [1]–[5]. Population management is crucial for several species, in particular to keep or increase genetic diversity of a population, e.g., to reduce inbreeding (e.g., Delacour's langur [*Trachypithecus delacouri*] in Vietnam [6]; grey wolf [*Canis lupus*] in the northern Rocky Mountains, United States [7]; and Siberian jay [*Perisoreus infaustus*] in Finland [8]). Important prerequisites for conservation and population management are information on population size, population dynamics, genetic diversity, genetic population structure, the mating system, and the current and historical distribution [6], [9]–[15].

Nearly 50% of the World's primate species are threatened (classified as vulnerable, endangered or critically endangered on the IUCN Red List [3]), among them the Guizhou snub-nosed monkey (*Rhinopithecus brelichi*). This endangered species is endemic to China and it occurs only in a single population of less than 900 individuals in the Fanjingshan National Nature Reserve (FNNR), Guizhou Province of southern China [16]–[20]. Compared to the closely related species *R. roxellana* and *R. bieti, R. brelichi* shows a low diversity of mitochondrial DNA (mtDNA) [21]. Since mtDNA is only maternally inherited, variation in this marker reflects only a part of the overall genetic variation within a population. A low diversity of mtDNA compared to related species describes only a low female effective population size (N_ef_) sometime in the past and does not give feasible information about the total genetic variation of the species. To get a better understanding of the genetic diversity of a population or species, a combined analysis of nuclear DNA (nDNA) and mtDNA diversity is more powerful. Since for *R. brelichi* only data on mtDNA diversity is available [21], we genotyped wild *R. brelichi* samples at eight microsatellite loci to gain information on nDNA diversity. Since the population size of *R. brelichi* is relatively small, we expect to find a similar impoverished nuclear genetic diversity as for mtDNA, which then would make a population genetic management plan more important [22].

## Materials and Methods

### Ethics statement

Research complied with protocols approved by the Forest Ministry of Guizhou Province, China, and adhered to the legal requirements of China and Germany, where research was conducted. Permits to collect samples were provided by the State Forestry Administration of China and Fanjingshan National Nature Reserve, and the staff of the nature reserve helped to collect samples and supported the study. The research was carried out in compliance with respective animal care regulations and the principles of the American Society of Primatologists and the German Primate Center for the ethical treatment of nonhuman primates. Fecal samples from wild individuals were collected non-invasively after the animals left the site without disturbing, threatening or harming them in their natural behavior.

### Field site and sample collection

**Figure 1:**
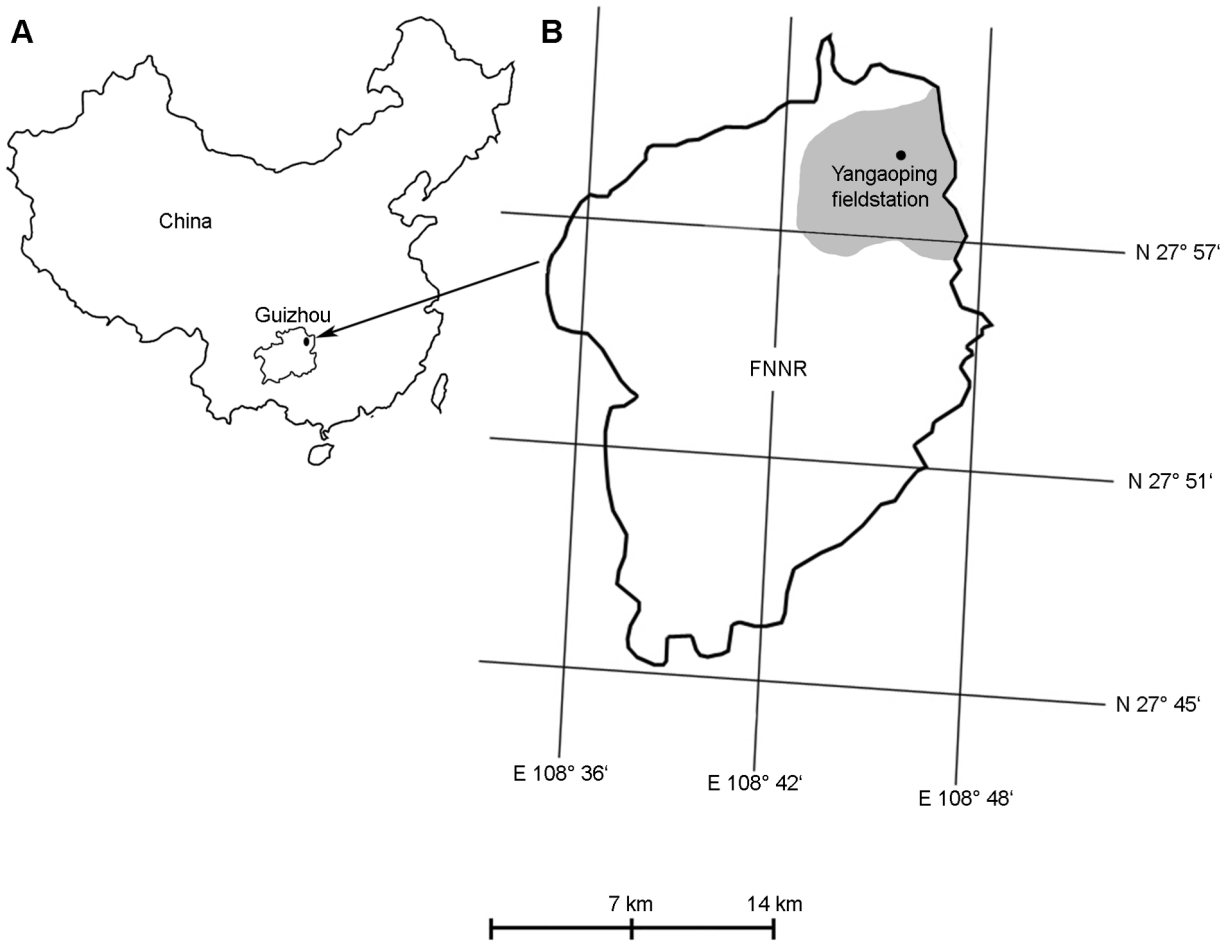
Geographical position of FNNR (marked by a black dot) in Guizhou Province, China (A) and a sketch of FNNR (B). Collection of samples for genetic analyses was carried out in the gray region around the Yangaoping field station.

The FNNR, established in 1986, covers the main peaks of the Wuling Mountains in Guizhou Province of southern China (27°45′10′′–28°02′24′′N, 108°35′03′′–108°48′23′′E, 480 – 2,750 m, Figure 1). Its size is just 38,300 ha (25,000 ha core zone, 13,300 ha buffer zone). About 15,000 people live in the buffer zone and about 1,100 in the core area of the reserve [19]. The vegetation in the area is subtropical, semi-evergreen mixed and evergreen deciduous forest. In the area also coniferous forest is present, but *R. brelichi* is rarely found there. The species occurs manly in secondary forest, because primary forest is almost completely gone [20].

The core distribution of the monkeys is in the northern part of FNNR close to the field station Yangaoping (27°58′54.37′′N, 108°54′36.04′′E, 1,627 m, Figure 1). About 400–500 monkeys live around the station [18], [19], [23]. In this area, a total of 146 *R. brelichi* samples were collected during surveys in 2007 and 2008 [21]. Details about sample collection are described in Yang et al. [21].

### Laboratory methods

DNA extraction followed methods outlined in Yang et al. [21]. To prevent contamination, all steps were performed in a sterile workbench (Captair® flow) and after each sample the workbench was cleaned with ethanol and decontaminated with UV-light. Concentration of DNA was measured on a NanoDrop® ND-1000 spectrophotometer (NanoDrop Technologies). All 146 samples were genotyped at eight microsatellite loci (D1S533, D2S1326, D6S264, D6S501, D7S2204, D8S505, D10S1432, and D17S1290) using fluorescent-labelled primers (FAM, TET, or TAMRA) (Table 1). PCR reactions were carried out in a total volume of 30 µl, including 18.6–19.6 µl H_2_O (HPLC quality), 3 µl 10x buffer (contains 15 mM MgCl_2_, Biotherm), 4 µl BT buffer (contains 10 mg/ml BSA, 0.7% Triton X-100), 1 µl of each primer (10 µM), 0.2 µl dNTPs (25 mM), 0.2 µl Taq DNA polymerase (Biotherm, 5 u/µl) and 1–2 µl (50–100 ng) DNA. The PCR profile was 2 min at 94°C; then 40 cycles of 30 seconds at 94°C, 30 seconds at varying annealing temperatures (Table 1), 30 seconds at 72°C; followed by 5 min at 72°C. PCR amplification success was checked by visualization of 1 μl of PCR product under UV light after electrophoresis on 1% agarose gels containing ethidium bromide. Concentration of PCR products was estimated by comparison with diluted concentration standards (Fermentas pUC19 DNA 0.5 µg/µl) of 5 ng/μl and 10 ng/μl, respectively. For analysis, 1–5 ng PCR product was taken and analyzed on an ABI 3130xL Genetic Analyser (Applied Biosystems) along with the Genescan™ 400 HD [Rox™] Size Standard and Hi-Di™ Formamid, according to manufactures instructions (8.775 µl Hi-Di™, 0.225 µl standard, and 1 µl PCR product). GeneMapper^®^ v3.7 software (Applied Biosystems) was applied to determine allele sizes and genotypes. To avoid false inferences, the genotyping was repeated multiple times following the approach proposed by Morin et al. [24]. For heterozygous at least three repetitions, and for homozygous three to seven repetitions were performed from independent DNA extractions. Since fecal samples contain mainly degraded and low amounts of nDNA, allelic dropout and null alleles could lead to incorrect genotypes [24]. Thus, Micro-Checker v2.2.3 [25] was applied to test for the presence of possible null alleles, large allelic dropout or scoring errors due to stuttering.

**Table 1:** List of microsatellite loci.

locus	repeat motif	T_a_ [°C]	label	allele no.	allelic range [bp]	primer sequences (5′–3′)
D1S533	tetra	52	FAM	5	200–216	F: CATCCCCCCCAAAAAATATA
						R: TTGCTAATCAAATAACAATGGG
D2S1326	tetra	58	FAM	3	198–206	F: AGACAGTCAAGAATAACTGCCC
						R: CTGTGGCTCAAAAGCTGAAT
D6S264	di	58	FAM	6	113–123	F: AGCTGACTTTATGCTGTTCCT
						R: TTTTCCATGCCCTTCTATCA
D6S501	tetra	58	FAM	4	157–171	F: GCTGGAAACTGATAAGGGCT
						R: GCCACCCTGGCTAAGTTACT
D7S2204	tetra	58	TET	7	235–261	F: TCATGACAAAACAGAAATTAAGTG
						R: GGGTTCTGCAGAGAAACAGA
D8S505	di	57	FAM	11	140–172	F: CAAAAGTGAACCCAAACCTA
						R: AGTGCTAAGTCCCAGACCAA
D10S1432	tetra	58	TAMRA	5	145–161	F: CAGTGGACACTAAACACAATCC
						R: TAGATTATCTAAATGGTGGATTTCC
D17S1290	tetra	52	FAM	10	216–258	F: GCCAACAGAGCAAGACTGTC
						R: GGAAACAGTTAAATGGCCAA

T_a_: annealing temperature.

### Microsatellite analysis

First, we tested if there is any population structuring in our data set using a Bayesian approach in STRUCTURE v2.3.4 [26]. Program settings were set to the admixture model and a total run length of 1,000,000 iterations, a burnin of 100,000 and values of *K* (assumed number of populations) from 1–3. GENPOP v4.0.10 was applied to test for linkage disequilibrium, using default settings (demomorization number: 1,000; number of batches: 100; number of iterations per batch: 1,000) [27], [28]. We used GenAlEx v6.5 [29] for calculating allelic range, observed and effective number of alleles, observed (H_o_) and expected (H_e_) heterozygosities, and fixation index (F_is_) to measure deviation from Hardy-Weinberg Equilibrium (HWE) based on heterozygosity excess, with significance of deviations from zero assessed through bootstrapping with 1,000 replications across loci to generate 95% confidence intervals [30].

A significant negative F_is_ value indicates heterozygous excess, contrarily, a significantly positive F_is_ value indicates heterozygous deficit (homozygote excess) in the population. Hamilton [30] suggested that a deficit of heterozygosity provides evidence for consanguineous mating taking place in a population, while heterozygosity excess indicates a reduction of population size.

To detect indication for a recent bottleneck or a reduction of population size in *R. brelichi*, we used Bottleneck v1.2.02 [31]. We performed the one-tailed Wilcoxon signed rank test to test for significant excess of heterozygotes. Piry et al. [31] suggest that the Wilcoxon test gives most reliable results when less than 20 polymorphic loci are studied. When a shrinking population gets small, the allele number is faster reduced than the expected heterozygosity. Thus, the heterozygosity excess (H_e_ > H_eq_: H_e_ based on allele frequencies; H_eq_: heterozygosity expected at mutation equilibrium, based on the number of alleles) was tested with the Two-Phase Model (TPM) [32], which combines the Infinite Allele Model (IAM) [33] with the Stepwise Mutation Model (SMM) [34]. The true model of mutation for most loci is between the IAM and SMM [35] and since the SMM is more suitable for microsatellite data than the IAM [36], we selected the TPM method with 90% single-step and just 10% multiple-step mutations. A significant heterozygosity excess (H_e_ > H_eq_) indicates a recent bottleneck event [36]. For the TPM, 10,000 iterations were applied. The variance was set to 12 as recommended [31]. We also applied a qualitative graphical method for detecting recent bottlenecks based on a mode-shift distortion of the allele frequency distribution in Bottleneck. Bottlenecks cause alleles at low frequency (<0.1) to become less abundant than alleles in one or more intermediate allele frequency class (e.g., 0.1–0.2) thus shifting the mode of the normally L-shaped frequency distribution into higher frequency classes [35], [37], [38].

### Mitochondrial DNA analysis

To infer timing and magnitude of past changes in *R. brelichi* N_ef_, we performed a Bayesian Skyline Plot (BSP) in BEAST v1.7.4 [39], [40]. Therefore, we used sequences of the hypervariable region I (HVI) of the mitochondrial control region published by Yang et al. [21]. In a total of 141 individuals (as determined by genotyping), only five haplotypes were found (HQ891105-HQ891109) with a frequency of the most dominant haplotype of 0.723 [21]. The haplotype network presented in Yang et al. [21] and the fact that there are obviously no physical barriers for primates in FNNR suggest that the *R. brelichi* population at FNNR is not sub-structured. Hence, false inferences from the BSP due to population structure are unlikely [41].

For the BSP, we applied the HKY model [42] as selected by the Bayesian Information Criterion in jModelTest v0.1.1 [43], [44]. The clock model was set to a strict clock with a generation time of 12 years according to a female age of first reproduction of 8–9 years, and a lifespan of about 18 years [45]. We used a substitution rate of 0.1643 substitutions per nucleotide per million years [21] and a coalescent Bayesian Skyline tree prior with 10 groups under a piecewise-constant skyline model. The length of chains was set to 50 million generations with logged parameters every 1,000 generations. Tracer v1.5.0 was applied to check chain convergence and to visualize the BSP.

## Results

### Microsatellites

Of the 146 collected samples, genotyping showed that they derived from 141 different individuals. We found no evidence for null alleles, large allelic dropout, scoring errors due to stuttering or sub-structuring in our samples. The observed number of alleles (N_a_), effective number of alleles (N_e_), observed heterozygosity (H_o_), expected heterozygosity (H_e_), and the fixation index (F_is_) are shown in Table 2. The N_a_ per locus ranges from 3 to 13 with a mean of 6.63±3.34. The mean N_e_ resulted in 3.84±1.43 ranging between 2.23 and 5.77. The mean H_e_ across eight polymorphic loci (0.70±0.12) is similar to the mean H_o_ (0.71±0.12). All values indicate that all eight microsatellite loci are highly polymorphic in the *R. brelichi* population (Table 2). The values for H_o_ and H_e_ for all microsatellite loci are not different (p = 0.85). Thus, no deviation from HWE is indicated. Similarly, the overall F_is_ value for the eight microsatellite loci is not significantly different from zero (p = 0.72) also suggesting that the *R. brelichi* population does not depart from HWE. However, D1S533 (F_is_ = 0.14) and D17S1290 (F_is_ = 0.22) show significant positive F_is_ values (p<0.05) indicating heterozygosity deficits. A test for linkage disequilibrium revealed no significant linkage pattern.

**Table 2:** Genetic parameters for eight microsatellite loci in *Rhinopithecus brelichi*.

Locus	N_a_	N_e_	H_o_	H_e_	F_is_
D1S533	5	2.23	0.48	0.55	0.14*
D2S1326	3	2.29	0.85	0.56	−0.51
D6S264	6	4.96	0.76	0.8	0.04
D6S501	4	3.26	0.77	0.69	−0.12
D7S2204	7	4.58	0.74	0.78	0.05
D8S505	13	5.77	0.81	0.83	0.02
D10S1432	5	2.48	0.65	0.6	−0.08
D17S1290	10	5.13	0.63	0.81	0.22*
mean	6.63	3.84	0.71	0.70	overall:
SD	3.34	1.43	0.12	0.12	p = 0.72

N_a_  =  no. of alleles; N_e_  =  no. of effective alleles; H_o_  =  observed heterozygosity; H_e_  =  expected heterozygosity; F_is_  =  fixation index; * = p<0.05.

A comparison of H_e_ and H_o_ values of *R. bieti* (data taken from Liu et al. [46]) and *R. brelichi* for six of our eight loci (D1S533, D2S1326, D6S264, D7S2204, D8S505, and D17S1290) revealed no significant differences between both species (*R. brelichi*: H_e_: 0.72±0.13; H_o_: 0.71±0.14; *R. bieti*: H_e_: 0.78±0.09; H_o_: 0.58±0.27; H_e_: t = −1.5, p = 0.19; H_o_: t = 0.94, p = 0.39; Table 3).

**Table 3:** Expected (H_e_) and observed (H_o_) heterozygosity across six loci for *Rhinopithecus brelichi* and *R. bieti* (data for *R. bieti* from Liu et al. [46]).

locus	H_e_ *R. brelichi*	H_e_ *R. bieti*	H_o_ *R. brelichi*	H_o_ *R. bieti*
D1S533	0.55	0.77	0.48	0.73
D2S1326	0.56	0.61	0.85	0.57
D6S264	0.80	0.87	0.76	0.07
D7S2204	0.78	0.77	0.74	0.69
D8S505	0.83	0.85	0.81	0.56
D17S1290	0.81	0.78	0.63	0.84
mean	0.72	0.78	0.71	0.58
SD	0.13	0.09	0.14	0.27

**Figure 2:**
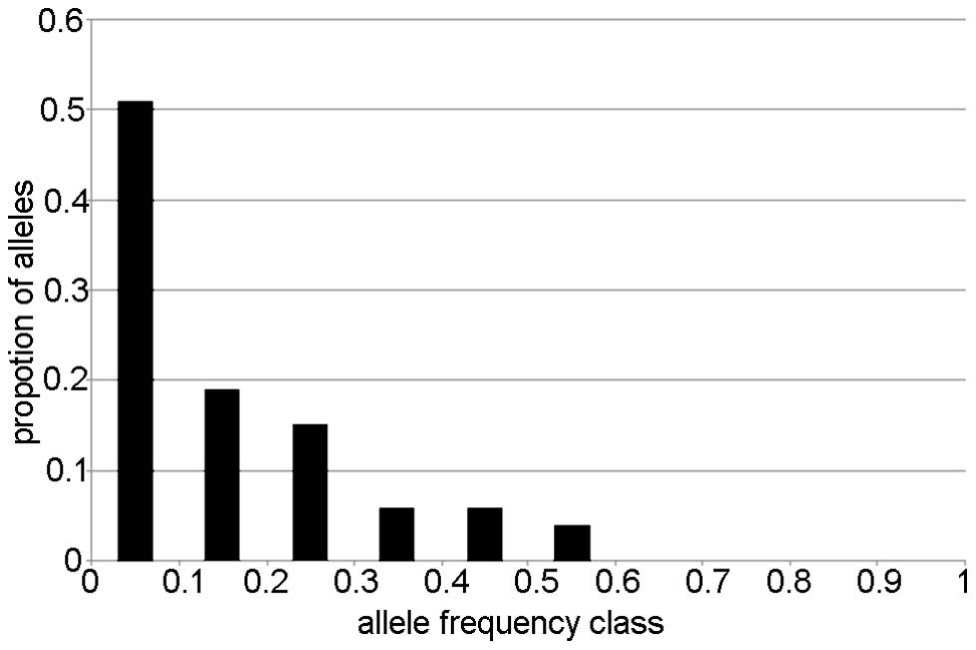
Allele frequency distribution for eight microsatellite loci in *Rhinopithecus brelichi* (n = 141 individuals). Bars represent the proportion of alleles found in each allele frequency class. The distribution is L-shaped, as expected for a stable population under mutation-drift equilibrium, thus not indicating a recent bottleneck.

The TPM implemented in the Bottleneck program does not support a recent bottleneck in the *R. brelichi* population (p = 0.098). Additionally the allele frequency distribution does not indicate any distortion form a normal L-shape distribution (Figure 2), hence also this graphic test does not support any recent bottleneck of the *R. brelichi* population.

### Mitochondrial DNA

**Figure 3:**
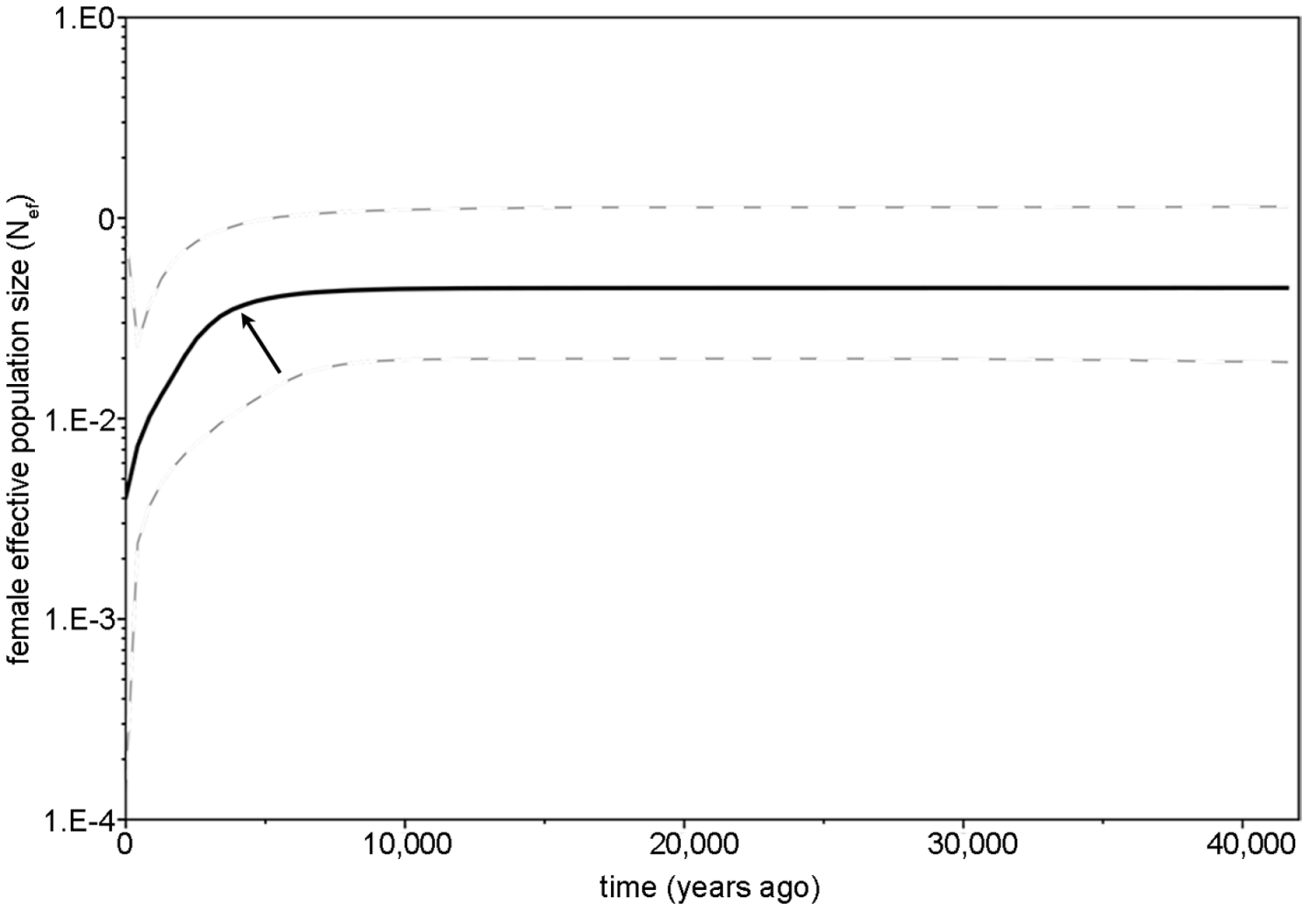
Bayesian Skyline Plot (BSP) displaying changes in female effective population size (N_ef_) through time in *R. brelichi*. Calculations are based on 603 bp of the mitochondrial HVI region. Shown are the median (black) and the 95% highest posterior probability density (dashed lines) around the estimate. The arrow indicates the start of a reduction in N_ef_.

To infer timing and magnitude of past changes in *R. brelichi* N_ef_, we performed a BSP using mitochondrial HVI region sequence data of the 141 individually assigned samples. The BSP (Figure 3) displays changes in the N_ef_ through time and indicates a reduction in population size between 3,500 and 4,000 years ago.

## Discussion

As shown by Yang et al. [21], *R. brelichi* (haplotype diversity [H]: 0.457±0.084; nucleotide diversity [π]: 0.014±0.007) exhibits a low diversity in mtDNA as compared to its congeners *R. roxellana* (H: 0.845±0.026; π: 0.034±0.017) and *R. bieti* (H: 0.948±0.006; π: 0.036±0.018). In contrast to the low diversity in mtDNA, diversity in nDNA does not differ from other *Rhinopithecus* species. All eight microsatellite loci were highly polymorphic as suggested by the high allelic richness (6.63±3.34) and heterozygosity (H_e_: 0.70±0.12; H_o_: 0.71±0.12). Similar allelic richness (7.5) and heterozygosity (H_e_: 0.70; H_o_: 0.61) were found in *R. bieti* [47] with a total population of less than 2000 individuals [3] and in *R. roxellana* (allelic richness: 4.5; H_e_: 0.59; H_o_: 0.59) [48] with a total population in Shennongjia Nature Reserve of approx. 500 individuals [49].

Since only one panmixing population of *R. brelichi* remained, we could not compare the genetic diversity among (sub-)populations to detect a loss of the overall genetic diversity, but we matched our results with respective data from *R. bieti* [46]. Both the expected and the observed rates of heterozygosity do not differ largely between the two species. Pan et al. [50] indicated that *R. roxellana* is highly polymorphic at microsatellite loci. Thus, with similar heterozygosity values, a similar high level of genetic diversity as in *R. roxellana* can be found in *R. brelichi* implying no indication for a reduction in terms of nDNA diversity in this species.

A heterozygosity deficit was detected at two of the eight loci: D1S533 (F_is_ = 0.14) and D17S1290 (F_is_ = 0.22). The deviation from HWE might be caused by null alleles or consanguineous mating taking place in this population [30]. However, the results from Micro-Checker do not show any evidence for null alleles. Since the population is relatively small and mating opportunities are limited, even more so if certain males are able to monopolize a number of females, on one hand consanguineous mating might happen frequently in *R. brelichi* and reduces their fitness (inbreeding depression) [51]. On the other hand *R. brelichi* could have a strong natural avoidance for inbreeding, which could lead to a decline of progeny and so to a decrease of population size [52].

The TPM provides no sufficient evidence for a recent bottleneck in the *R. brelichi* population. With a sample size of more than 15% of the total population we can exclude the weakness of Bottleneck tests caused by limited sample sizes [53]. Similarly false results caused by deviation from HWE are unlikely [35]. In the analysis of allele frequencies we found the most abundant alleles in the lowest frequency class (<0.1). The overall distribution of allele proportions in various frequency classes follows a normal L-shape (Figure 2). This supports the assumption that no bottleneck occurred in the recent history of *R. brelichi*. The high genetic diversity of nDNA and lack of evidence for a recent bottleneck strongly suggests that the *R. brelichi* population was in equilibrium during its recent history.

The analysis of mtDNA draws another picture. Although the BSP suggests a stable female effective population size (N_ef_) in *R. brelichi* over at least 25,000 to 35,000 years, a strong decline is indicated 3,500 to 4,000 years ago (Figure 3). A similar population trend was found in *R. bieti* [47] and *R. roxellana* [54]. According to geographic provenances of fossils and historical records, all snub-nosed monkey species in China were once widespread. Simultaneously with the decline of N_ef_ in the three Chinese snub-nosed monkeys, the human population increased and with it, significant modifications of the environment due to agricultural practices started, thus largely reducing suitable habitat for snub-nosed monkeys in China [55], [56].

### The obvious contrast

**Figure 4:**
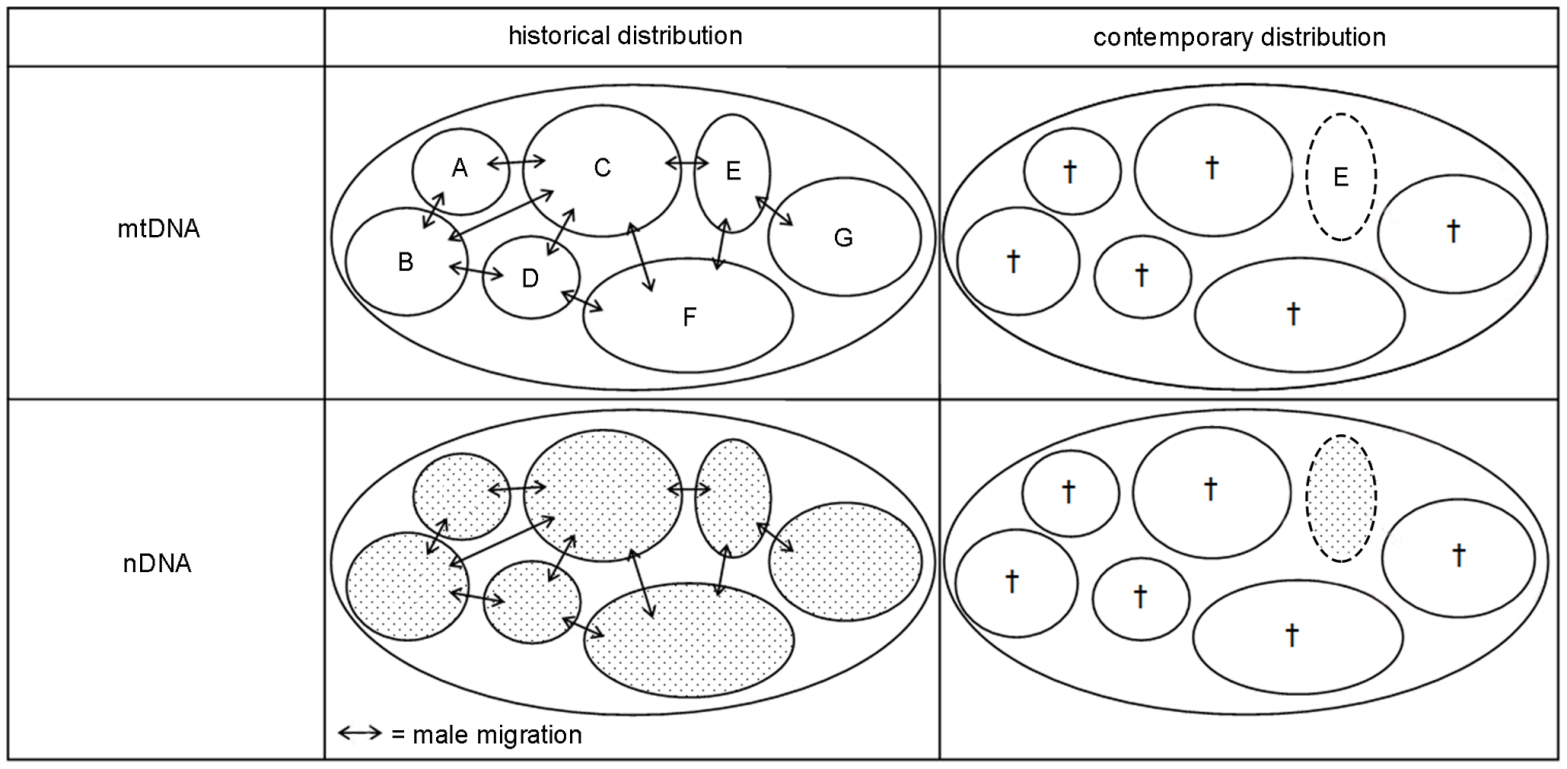
Scenario explaining discrepancies between mtDNA and nDNA diversity in *R. brelichi*. In historical times, *R. brelichi* was distributed over a larger area than today comprising several subpopulations or demes (A–G) with respective different mtDNA haplogroup assemblages. Due to male migration between these demes, nDNA was transferred between them and equalized nDNA diversity among demes, but not so for mtDNA. After partial habitat and population loss in this example only one deme survived (E; dashed circles), containing just a subset of the original mtDNA haplotypes but almost all nDNA diversity. Thus, mtDNA diversity was strongly reduced while nDNA diversity remained comparatively high.

High nDNA diversity combined with low mtDNA diversity is uncommon and not often observed in mammals. One example is the Scandinavian brown bear (*Ursus arctos*), which was close to extinction in the 1930s [57]. In the bear population, the nDNA diversity is much larger than the mtDNA diversity [57]. Waits and colleagues [57] suggested as a possible reason for this discrepancy the greater maximum dispersal distances of male bears in contrast to females. This would lead to a localized distribution of mtDNA haplotypes but a broader geographic distribution of nDNA variants. A similar scenario can be envisioned for *R. brelichi*. In historical times, when the species had a much larger distribution with several demes [56], female philopatry and male migration would contribute to a mtDNA population structure consisting of local populations with certain haplotype assemblages, since males do not transfer their mitochondria. On the nDNA level however, male migration would contribute to gene flow and a population with no or only a weak spatial genetic structure (Figure 4). After the extinction of almost all demes, many mtDNA haplotypes were lost and only those few in the surviving deme remained. In contrast, the remaining population would nevertheless comprise a large proportion of the former nDNA diversity (Figure 4).

Our results suggest that the overall genetic diversity of the endangered R. brelichi is not reduced to a degree that it might become a direct threat for the long-term survival of the species. In contrast, due to the very limited distribution and population size of *R. brelichi*, conservation strategies should be focused on direct threats to this species (such as tourist infrastructure development, deforestation, grazing of livestock, using the reserve as source for food and fire wood) and try to remove or limit them. An enlargement of the reserve would be advantageous, but it seems even more important to establish at least one additional, but geographically isolated population [3]. A translocation of some individuals into another habitat might minimize the risk that a stochastic event like a forest fire or a disease could lead to extinction of the species. There could be several sites within the Wuling Mountains that might offer such habitats [3], but thorough surveys and habitat suitability analysis are required to find them.
